# Benchmarking four commercial antimicrobial susceptibility testing systems and disk diffusion to reference broth microdilution in the assessment of gram-negative bacterial isolates

**DOI:** 10.1128/jcm.00502-26

**Published:** 2026-09-09

**Authors:** Daniel D. Rhoads, Jingzi Sherman, Felicia Chen, Kristin Baker, Karissa Culbreath, Erin McElvania, Amanda Harrington, Vincent Streva, Jamie Marino, Lars F. Westblade, Eric Stern, Patricia J. Simner

**Affiliations:** 1 Pathology & Laboratory Medicine Department, Cleveland Clinic, Cleveland, Ohio, USA; 2 Microbial Sciences in Health Department, Cleveland Clinic Research, Cleveland Clinic, Cleveland, Ohio, USA; 3 Department of Pathology, Cleveland Clinic Lerner College of Medicine, Case Western Reserve University School of Medicine, Cleveland, Ohio, USA; 4 Selux Diagnostics, Charlestown, Massachusetts, USA; 5 Department of Infectious Disease, Tricore Laboratories, Albuquerque, New Mexico, USA; 6 Department of Pathology, University of Chicago, Pritzker School of Medicine, Chicago, Illinois, USA; 7 Department of Pathology and Laboratory Medicine, Loyola University, Chicago, Illinois, USA; 8 Division of Infectious Disease Diagnostics, Northwell Health Laboratories, New York, New York, USA; 9 Department of Pathology and Laboratory Medicine, Weill Cornell Medicine, New York, New York, USA; 10 Department of Laboratory Medicine and Pathology, Mayo Clinic, Rochester, Minnesota, USA

**Keywords:** antimicrobial resistance, antibiotic susceptibility testing

## Abstract

Commercial antimicrobial susceptibility testing (AST) systems for gram-negative bacteria provide operational advantages over reference broth microdilution (BMD) testing, but the results produced by commercial AST systems might not be as accurate as the reference method. Assessing and comparing the performance of AST systems can be informative for clinical microbiology practice. In total, 280 clinical isolates and 100 isolates from the U.S. Centers for Disease Control and Prevention Antimicrobial Resistance Isolate Bank, including Enterobacterales and *Pseudomonas aeruginosa,* were intended to be tested using disk diffusion (DD) and automated commercial AST systems, such as Selux AST System (Selux Diagnostics), MicroScan (Beckman Coulter), Phoenix (Becton, Dickinson and Company), and Vitek 2 (bioMérieux). These results were compared to BMD as the reference standard. In total, 372 isolates were assessed. For Enterobacterales, the greatest frequency of very major errors was identified with cefepime by Vitek 2 (29.0%) and ertapenem by DD (9.1%); essential agreement less than 90% was identified with the following combinations: aztreonam by MicroScan (86.4%), aztreonam by Vitek 2 (88.9%), cefepime by Selux (86.3%), cefepime by Vitek 2 (76.8%), ceftazidime by Vitek 2 (89.9%), and levofloxacin by MicroScan (83.4%). For *P. aeruginosa*, essential agreement less than 90% was identified with the following combinations: gentamicin by Selux (87.2%), tobramycin by Selux (86.6%), and ciprofloxacin by Phoenix (88.4%). Overall, antimicrobials that had low demonstrable bias across all automated commercial AST systems included ampicillin, amoxicillin-clavulanate, ceftazidime-avibactam, ertapenem, and trimethoprim-sulfamethoxazole. Comparing commercial testing systems head-to-head against a reference standard provides insight into the strengths and weaknesses of the performance of these test systems, and these insights can subsequently inform local decisions regarding further investigations and potential operational mitigation strategies.

Antimicrobial susceptibility testing (AST) is an important but challenging endeavor for clinical microbiology laboratories. Providing rapid and accurate AST can improve patient outcomes and antimicrobial stewardship (1, 2). However, providing rapid and accurate AST is challenging for many reasons. Even tests that are considered reference methods (e.g., fresh or frozen BMD) or standard methods (i.e., disk diffusion [DD]) have substantial accuracy and precision variability. Many clinical microbiology laboratories do not routinely use reference or standard methods, but they employ commercial AST systems for routine use because these systems can provide more rapid and operationally efficient results. However, commercial methods may have added performance limitations due to the compounding of biological and methodological variables.

In addition to the challenge of creating timely and accurate results, maintaining current clinical breakpoint interpretation criteria is an ongoing challenge. AST systems, cleared for use by the U.S. Food and Drug Administration (FDA), may be using obsolete breakpoints that are no longer recognized by the FDA, and the systems’ performance with contemporary breakpoints might not be routinely updated or assessed (3).

Additionally, AMR continues to evolve, and increased prevalence of resistance and novel mechanisms of resistance (e.g., carbapenemases) have emerged in recent decades (4). It is important to assess and monitor the performance of AST methods against the contemporary landscape of AMR in order to determine if the systems continue to achieve adequate accuracy.

To assess the current state of the performance of commercial AST *in vitro* diagnostic devices (IVDs) using contemporary clinical pathogens, the National Institutes of Health (NIH) funded the 5SB1AI136125–05 proposal: “Next-generation phenotypic antimicrobial susceptibility test platform: rapid results and comprehensive menus.” Data shared in this report represent some of the efforts of that study. The study compared the performance of a selection of commercial AST systems against the reference standard triplicate broth microdilution and the standard method of DD testing using gram-negative (GN) clinical isolates and multidrug-resistant (MDR) challenge isolates. The performance of the commercial AST systems was evaluated using the FDA susceptibility test interpretive criteria, including essential agreement (EA), categorical agreement (CA), minor errors (MIN), major errors (MAJ), very major errors (VMJ), bias, and the current (2024) FDA clinical breakpoints. The FDA breakpoints were identical to the Clinical and Laboratory Standards Institute (CLSI) breakpoints, except for cefoxitin, gentamicin, and piperacillin-tazobactam for Enterobacterales, and tobramycin and gentamicin for *Pseudomonas aeruginosa* (5) (Table S1).

## MATERIALS AND METHODS

### Multicenter study design

This study was conducted at four clinical laboratories in the United States between September 2022 and January 2024. Testing using four automated commercial AST systems with relevant accompanying panel(s) was performed: MicroScan WalkAway with the NM56 panel (Beckman Coulter; Brea, California), Phoenix with the NMIC-306 Emerge panel (Becton, Dickinson and Company [BD]; Franklin Lakes, New Jersey), Vitek 2 with the GN79 and XN15 panels (bioMérieux; Marcy-l'Étoile, France), and Selux AST system with the GN panel (Selux Diagnostics; Charlestown, Massachusetts). Manual DD testing was also performed as a standard method using disks manufactured by BD. These tests were compared to triplicate BMD testing as the reference method. All isolates underwent testing using each of the systems or methods. Each laboratory tested the isolates using one or two AST systems available in the local laboratory.

### Bacterial isolate selection

A total of 380 frozen GN bacterial isolates were distributed to the laboratories for testing and were intended to be used in the study. The isolates included 280 clinical isolates from the past clinical trials conducted by Selux Diagnostics, which Selux shipped to the laboratories, and 100 isolates from the U.S. Centers for Disease Control and Prevention (CDC) Antimicrobial Resistance (AR) Isolate Bank, which the laboratories acquired directly from the CDC with various mechanisms of antimicrobial resistance. The tested isolates (Table 1) included 130 *P. aeruginosa* and 242 Enterobacterales from 11 taxa: *Citrobacter freundii* complex*, Citrobacter koseri, Enterobacter cloacae* complex*, Escherichia coli, Klebsiella aerogenes, Klebsiella oxytoca, Klebsiella pneumoniae, Morganella morganii, Proteus mirabilis, Proteus vulgaris,* and *Serratia marcescens*. The 280 clinical isolates were chosen from a bank of clinical isolates that were collected during a randomized clinical trial that spanned from 2020 to 2022 (6). Isolates were considered for the present study if they had valid BMD results, and the sample of isolates was selected by enriching for resistant isolates and diversity of taxa. The 100 CDC AR Isolate Bank isolates exhibited a variety of resistance mechanisms and patterns across multiple CDC AR panels: *P. aeruginosa*, Enterobacterales Carbapenemase Diversity (CRE), ceftazidime-avibactam (CZA), imipenem-relebactam (IMR), meropenem-vaborbactam verification (MEV), and the Multi-site Gram-negative Surveillance Initiative (MuGSI). The CDC AR bank isolates had CDC-published reference modal minimum inhibitory concentration (MIC) results that were confirmed by reference BMD at an independent reference laboratory. Isolates that demonstrated inconsistent identifications/morphologies or BMD results inconsistent with the CDC’s reported results were excluded from the study.

### AST

Each frozen isolate was sub-cultured from a frozen state to a 5% sheep blood tryptic soy agar plate (F1), followed by a second subculture (F2) before testing. Quality control was performed according to standard laboratory procedures, which included following guidance in the instructions for use (IFU) and individual quality control plans (as defined by the Centers for Medicare and Medicaid Services). In some instances, isolate testing was repeated, and the results were replaced when sampling preparation or operational errors were identified, purity plates showed mixed growth, or when the results were not obtained with initial testing. If a system did not provide the results for a specific antimicrobial-organism combination (e.g., suppressed results due to system limitations), the results were not included in the analysis. Local laboratory reporting rules were not applied to the results.

Isolate testing followed existing routine laboratory procedures. For MicroScan users, Prompt Inoculation System (BD Part B1026–10D) was used routinely; all *S. marcescens* isolates were tested using the turbidity method due to regulatory claims that limit the use of *Serratia* with the Prompt Inoculation System (https://www.accessdata.fda.gov/cdrh_docs/reviews/K202343.pdf). A single determination was obtained for each isolate using the commercial AST systems. For Enterobacterales, the species evaluated for each antimicrobial agent is listed in Table S2, except for specific cases. For the MicroScan system, *E. cloacae* complex and *P. vulgaris* isolates were not assessed for susceptibility to ceftazidime-avibactam, while *C. freundii* complex and *P. vulgaris* isolates were not tested for meropenem-vaborbactam. In the case of the Phoenix system, *P. vulgaris* isolates were not tested for susceptibility to ampicillin-sulbactam, and *P. mirabilis* isolates were not evaluated for meropenem. No results were collected for these specific cases. The lack of susceptibility assessment was due to the laboratory’s response to a pre-study questionnaire regarding which antimicrobial-organism combinations are validated, tested, or offered in routine practice using the lab’s AST system, as the intention of the study was to assess the performance of testing that was available in the local laboratories.

Except for Selux testing, the labs performing testing used test systems with which they were familiar and which were used locally and routinely for standard of care testing at their locale (Table 2). Selux testing was performed in a clinical environment, but the test system was less familiar to the testing team, as the platform was not used routinely for standard of care testing.

Antimicrobials that could be tested by all methods were intended to be included in the study. Antimicrobials, reportable MIC ranges, and clinical breakpoints applied for the commercial AST systems are found in Fig. 1 and 2 and in Table S3. The MIC results were recorded for all four commercial AST systems. The MIC results reported by the systems were considered, but method-specific expert rules for interpretation of the results were not considered. DD was performed following CLSI M02 guidelines (7) and CLSI M100 34th edition (5).

### Reference methods and results

Reference BMD testing for the clinical isolates followed CLSI M07 (8). Triplicate BMD was performed by JMI Laboratories (North Liberty, Iowa) using three separate inocula. When triplicate results had mode or median results with essential agreement, the mode or median MIC was used as the reference result. For example, the mode was used if there were two identical results, and in the absence of a mode, the median result was used. In cases where these criteria were not met (<10% occurrence), two additional replicates were tested from separate inocula, and the median MIC of the five determinations was used. Quality control was performed on each testing day. Reference results for the CDC AR isolates were based on the published results from CDC (https://wwwn.cdc.gov/arisolatebank/).

### Performance characteristic evaluation

All isolates with results that were generated by the AST system were included in the analysis. For data analysis, *P. aeruginosa* and the 11 Enterobacterales taxa were grouped together and individually. Essential agreement (EA), categorical agreement (CA), and categorical errors were analyzed according to the 2009 FDA Class II Special Controls Guidance Document: Antimicrobial Susceptibility Test Systems (2). Briefly, EA was defined as the number of MIC results within one doubling dilution of the reference results; CA was defined as the number of testing results with the same categorical interpretation (e.g., susceptible [S], intermediate [I], or resistant [R]) as the interpretation of the reference result. Both EA and CA rates were calculated using the total number of isolates as the denominator. Error rates were defined as VMJ (false susceptible), MAJ (false resistant), and MIN (SDD [susceptible-dose-dependent] or I by one method but either S or R by the other). All errors were determined by comparing to BMD as the reference and by applying FDA Susceptibility Test Interpretive Criteria breakpoints from January 2024 (Fig. 1 and 2 and Table S3). VMJ rates were calculated using the number of isolates resistant by BMD as the denominator, and MAJ rates were scored using the number of isolates with BMD results that were susceptible as the denominator. Each commercial system produced a different number of on-label or valid AST results due to the manufacturer-suppressed results, resulting in a different number of isolates being assessed for each system’s antimicrobial-organism combination. Thus, the denominators for all calculations varied according to the number of valid results obtained from each system. The results obtained using the DD method only contained categorical interpretations; therefore, the EA was not calculated.

Generally, the FDA’s approach to performance expectations is for EA and CA to be >89.9%, VMJ not to exceed 2%, MAJ not to exceed 3%, and assessment of bias (9). Bias analysis was performed according to the FDA’s definition. Only evaluable isolates were included in the analysis. The evaluable sample results were defined as any result that either (i) had on-scale MIC values for both the reference and the investigated AST method or (ii) had an on-scale value for either the reference or test, which was at least one doubling dilution from the off-scale result. For example, an investigated AST test result of ≤2 μg/mL and a reference result of 4 μg/mL were considered evaluable and within EA, but a result of ≤2 μg/mL for both the investigated AST test and the reference would not be considered.

### Time-to-result evaluation

The time-to-result duration was calculated from the initiation of sample processing to the availability of results for each sample and subsequently categorized by the GN organism groups for each commercial system. For the DD method, the duration was calculated from the commencement of sample preparation to the point at which results were interpreted by the operator.

## RESULTS

Of the 380 isolates that were intended to test, eight (2.1%) were excluded from the data analysis, including one AR bank isolate and three clinical isolates that subsequently were determined to have discrepant identities to that which was recorded, one clinical isolate exhibiting multiple morphologies, and three AR bank isolates with BMD results that were discrepant with the CDC-published reference results (Table 1). Thus, a total of 372 isolates had evaluable AST results that will be described in this study (Table 3). The total numbers of organism-antibiotic tests assessed in this study for Selux, MicroScan, Phoenix, Vitek, and DD were 3,872, 3,869, 3,909, 3,513, and 3,571, respectively (Table S4). Differences in the total number of organism-antibiotic tests between systems are due to the limitations of claimed organism-antibiotic combinations by the manufacturer, local reporting practices as described in Materials and Methods, or invalid results. The number of invalid organism-antibiotic tests was not recorded. More details on specific organism-antibiotic tests per commercial AST system can be found in Table S4.

For Enterobacterales (Table 4), CA less than 90% was identified in the following test combinations: cefoxitin by Selux (75.2%), cefoxitin by Vitek 2 (81.6%), cefoxitin by DD (76%), cefepime by Vitek 2 (75.9%), cefepime by DD (84.3%), ceftazidime by MicroScan (89.8%), levofloxacin by DD (87.6%), minocycline by MicroScan (81.8%), minocycline by Vitek 2 (74%), and minocycline by DD (83%). EA less than 90% was identified in the following test combinations: aztreonam by MicroScan (86.4%), aztreonam by Vitek 2 (88.9%), cefepime by Selux (86.3%), cefepime by Vitek 2 (76.8%), ceftazidime by Vitek 2 (89.9%), and levofloxacin by MicroScan (83.4%). The greatest frequency of MAJ was identified with minocycline by MicroScan (5.8%), levofloxacin by MicroScan (5.4%), and piperacillin-tazobactam by Phoenix (8.2%). The greatest frequency of VMJ was identified in cefepime by Vitek 2 (29.0%), ceftazidime by Vitek 2 (6.0%), ceftazidime by DD (5.0%), ertapenem by DD (9.1%), and levofloxacin by DD (5.1%).

For *P. aeruginosa* (Table 5), CA was less than 90% for the following combinations: gentamicin by MicroScan (86.9%), gentamicin by Phoenix (89.9%), and ciprofloxacin by Phoenix (82.9%). EA less than 90% was identified for the following test combinations: gentamicin by Selux (87.2%), ciprofloxacin by Phoenix (88.4%), and tobramycin by Selux. Phoenix demonstrated 4.9% MAJ and 4.7% VMJ for ciprofloxacin, and Phoenix had 18% VMJ for amikacin.

Bias trends are described in Table 6. The number of isolates included in the bias calculations differed substantially across the commercial AST systems due to differences in on-panel dilutions (Fig. 1 and 2). Most antimicrobials demonstrated positive or negative bias of at least 30% with 95% confidence by at least one method. Antimicrobials that exhibited at least 30% bias with 95% confidence in at least three of the four commercial AST systems included amikacin, cefazolin, ceftriaxone, ciprofloxacin, gentamicin, and meropenem. Antimicrobials that had acceptable demonstrable bias across all systems included ampicillin, amoxicillin-clavulanate, ceftazidime-avibactam, ertapenem, and trimethoprim-sulfamethoxazole.

The times required to obtain results using the different test systems are reported in Table 7. The median time from set-up to result for the commercial AST systems was 5.8–16.3 h for Enterobacterales and 7.2–16.3 h for *P. aeruginosa*. Selux had the shortest median time to results (6–7 h), which was more than five hours briefer than the next fastest method, which was Vitek 2 (11–14 h). MicroScan and Phoenix had similar median time to results (15–16 h), and DD took the longest (25 h).

## DISCUSSION

To our knowledge, this study is the broadest and largest comparison of GN AST systems using reference BMD testing (triplicate testing) as the reference method. The study included four commercial MIC AST systems, including MicroScan, Vitek 2, and Phoenix, which are widely used in the United States (3), and one recently FDA-cleared test system, Selux, which was recently sold through a public auction (10). A handful of rapid AST systems recently received FDA clearance, including Selux, and a detailed assessment of the performance of these new systems is needed. Finally, the study included DD testing, which until recently was considered a reference method for AST and is a commonly used method in clinical laboratories. Some of the largest errors identified in this study were challenges with Phoenix testing ciprofloxacin against *P. aeruginosa* and Vitek 2 testing cefepime and ceftazidime against Enterobacterales. These weaknesses could potentially be mitigated by using laboratory-based expert rules that require harmony of fluoroquinolone interpretations or on-instrument expert rules (11, 12). For example, ciprofloxacin and levofloxacin interpretations are typically concordant in *P. aeruginosa* (13); hence, investigating discordant interpretations could be a reasonable intervention. Vitek 2’s weak cefepime performance with Enterobacterales has been previously noted to be able to be mitigated by using the system’s Advanced Expert System (AES) software to alert the user of an unusual result and suspected error (11). Additionally, a high frequency of minor errors (>10%) was detected using DD for specific antibiotics: ampicillin-sulbactam, amoxicillin-clavulanate, aztreonam, cefepime, cefoxitin, and minocycline, which suggests that changes in disk breakpoints and/or disk potency could be further investigated (14).

Rapid AST systems are entering the U.S. market (15). Accelerate Pheno was the first of these systems, which first received FDA approval in 2017 and is limited in use to positive blood culture broth testing, but as of the time of this writing, the company has filed for bankruptcy and is no longer manufacturing. Later, in 2023 and 2024, several IVD platforms received FDA clearance, including Selux NGP (included in this study), bioMérieux Reveal, Q-Linea ASTar, and Affinity Biosensors LifeScale (15). A detailed assessment of the performance and experience of these new systems is needed. Some of these platforms and many additional commercial AST systems that are in development or in clinical trials are poised to drive down the AST turnaround times for isolates directly from positive blood culture broths and for bacterial isolates recovered in culture, and faster AST turnaround times can facilitate more rapid antibiotic optimization (16). The decrease in turnaround times can be 10 h or more compared to the current standard of care (Table 7). We expect that laboratories will begin adopting or may completely convert to rapid AST over the coming decade due to the substantial decrease in turnaround time and potential decrease in rapid AST cost in part due to market competition.

Reporting results from benchmarking studies, such as this study, which compare FDA-cleared IVDs that are competing in the market, can be informative and actionable for clinical laboratories. The information from these studies can identify strengths and weaknesses of different test systems, and these findings can aid laboratories in establishing high-value strategies and testing workflows that focus scrutiny and mitigation efforts on results known to have accuracy limitations. Laboratories can also combine the accuracy findings from benchmarking studies prior to implementing a given AST system together with local antibiogram data to determine which results may have low post-test probability of being accurate and focus risk mitigation efforts on these results (17).

Commercial test systems each have their own device-specific limitations described in their IFUs. These limitations are largely determined by the data submitted to the FDA during the initial clinical trials of the devices, but it can be challenging to assess the confidence of these stated limitations. Additionally, the approach to regulatory agencies can change over time, and in our experience, stringency of regulatory requirements tends to increase over time. Testing limitations declared in IFUs impacted this study; for example, piperacillin-tazobactam performance with *P. aeruginosa* was not assessed because one of the systems had an IFU-stated limitation for this organism-antibiotic combination. In clinical practice, mitigation of the IFU-stated limitations may require double-checking the AST result by using a different method or may require sending the isolate to a reference laboratory. Laboratories should be careful not to overlook these IFU limitations, but laboratories should attempt to proactively manage them. An important mitigation strategy that many commercial AST systems and laboratories use is expert rules that identify unusual results or detected mechanisms of resistance (e.g., extended-spectrum β-lactamases), and typically, these rules involve using more than only a single antimicrobial’s MIC to determine susceptibility or resistance (18). Although the variables triggering these alerts are not always transparent to the user, these expert rule systems can be an important part of quality management, but assessing their use and impact was outside the scope of this study, which focused only on the performance of the AST.

In addition to assessing the traditional metrics of EA, CA, VME, MAJ, and MIN, this study also evaluated the bias (Table 6) of the commercial MIC methods, where positive bias indicates the MIC of the test system trends higher than the reference MIC result, and negative bias indicates the MIC of the test system trends lower than the reference MIC result. Bias is now assessed by FDA, and bias assessment of commercial AST systems is also recommended by ISO 20776–2 (2021). The FDA and ISO bias calculation methods are not harmonized. The FDA limits evaluation to on-scale results, and the FDA’s approach was used in the current study. Although bias has not traditionally been a focus of MIC comparison evaluations, the authors agree that it is an important variable that can provide insights into systemic trends, which ultimately could impact the accuracy of susceptibility interpretations. Bias may play an important role when assessing the ability to update a clinical breakpoint on a system (3), and bias might also provide insights into which of two methods is most likely to be accurate when investigating unusual results. For example, based on the results of this study, many commercial systems had a positive bias when testing ciprofloxacin and *P. aeruginosa*, but disk testing agreed well with the reference method. Therefore, confirming the susceptibility of *P. aeruginosa* isolates that test “intermediate” to ciprofloxacin using a commercial system by retesting the isolate using disk diffusion could be a reasonable mitigation strategy to avoid overcalling resistance.

This study has a number of limitations that the reader should consider. First, expert rules are used by many commercial AST systems, and many laboratories employ additional custom expert rules, which can mitigate errors. However, expert rules were not used in this study (18). Second, combinations of a specific antibiotic and a specific taxon are noted in some IFUs as limitations, and these results were omitted from analysis in this study. These IFU-stated limitations will add workflow complexity in routine testing (Table S2). Alternatively, ignoring or overlooking these limitations and using a test system in an off-label manner may decrease the accuracy of the test system. Third, although this was a multicenter study, each isolate was tested by each method only once and only at a single laboratory. Intra-laboratory and inter-laboratory reproducibility were not evaluated in this study. Fourth, differences in dilution ranges and agents included on commercial AST panels limit the quality of comparisons across testing systems, especially for bias calculations. Fifth, a notable omission is piperacillin-tazobactam tested against *P. aeruginosa*, as the combination is not FDA-cleared for the Selux platform. Sixth, this study is a snapshot in time. Some AST panels evaluated during this study may be discontinued by the manufacturer in the near future. Also, breakpoint interpretations may change, which could impact the CA of the systems. For example, recent FDA susceptibility test interpretive criteria (STIC) breakpoint updates were not applied in this analysis, including the alignment of piperacillin-tazobactam breakpoints for *P. aeruginosa* with CLSI and updated aminoglycoside breakpoints that harmonized with CLSI breakpoints. Also, 2024 breakpoints were used for the assessment, which may be different than the original breakpoints used to initially assess the performance of the methods during regulatory trials. Additionally, the biology of AMR mechanisms continues to evolve, and the frequency of encountering AMR changes over time.

In conclusion, this study provides insights into the performance of several commercially available AST systems and methods for testing Enterobacterales and *P. aeruginosa*. A laboratory team can use this study’s findings to inform its local quality assurance practices, its expert rules, and/or its internal performance assessment of its AST system.

## Supplementary Material

Supplemental Tables S1 to S4

Supplemental Material

**Supplemental tables (JCM00502–26-S0001.docx).** Tables S1 to S4.

## Figures and Tables

**FIG 1:**
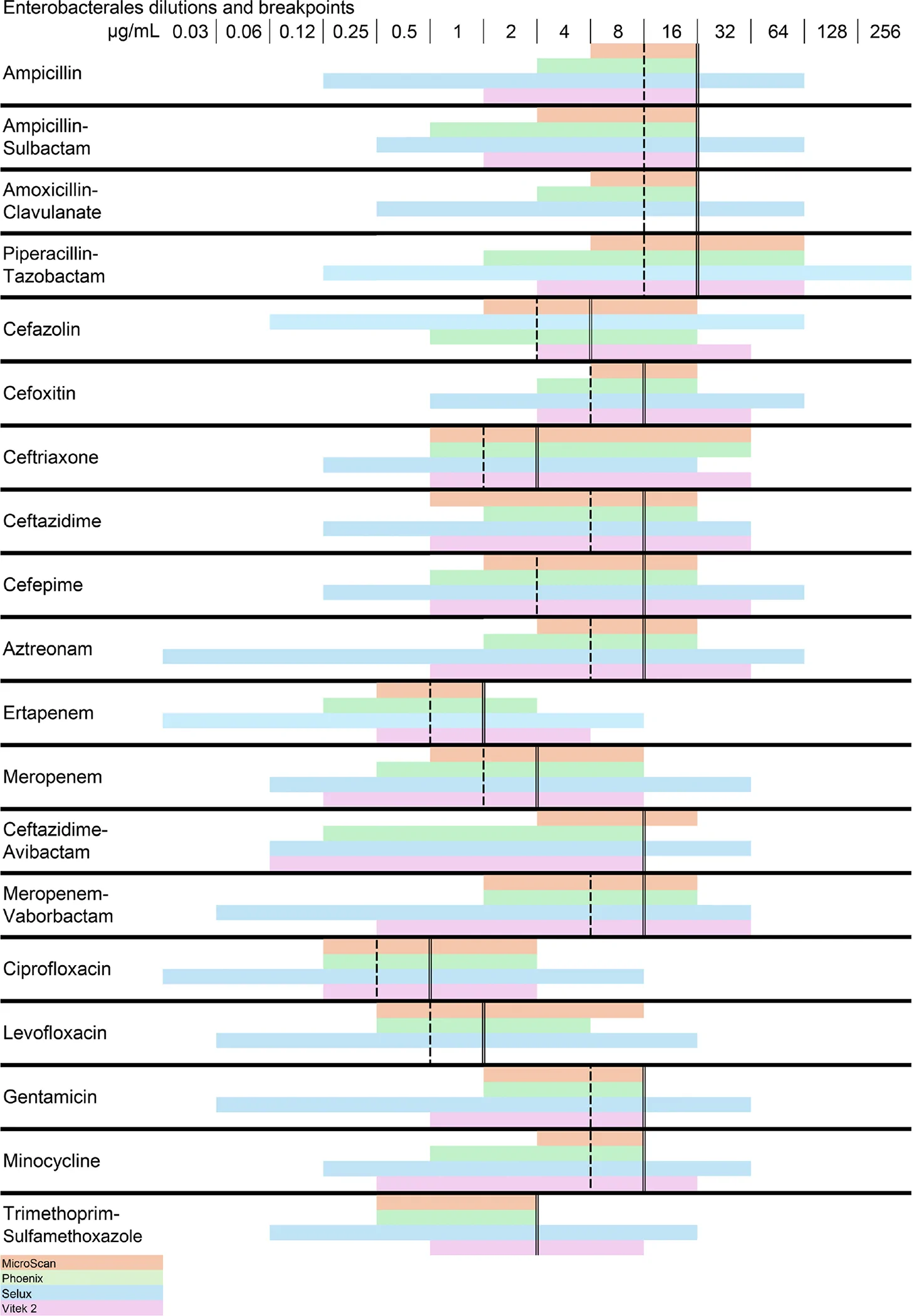
Tested antimicrobial dilutions and breakpoints for Enterobacterales. Dilutions on each panel are depicted on the grid by colored boxes. The 2024 U.S. FDA susceptibility testing interpretative criteria for Enterobacterales are depicted, where hashed lines demonstrate the breakpoint between susceptible and intermediate/susceptible-dose-dependent categories, and a double solid line demonstrates the breakpoint between resistant and intermediate (or between resistant and susceptible).

**FIG 2:**
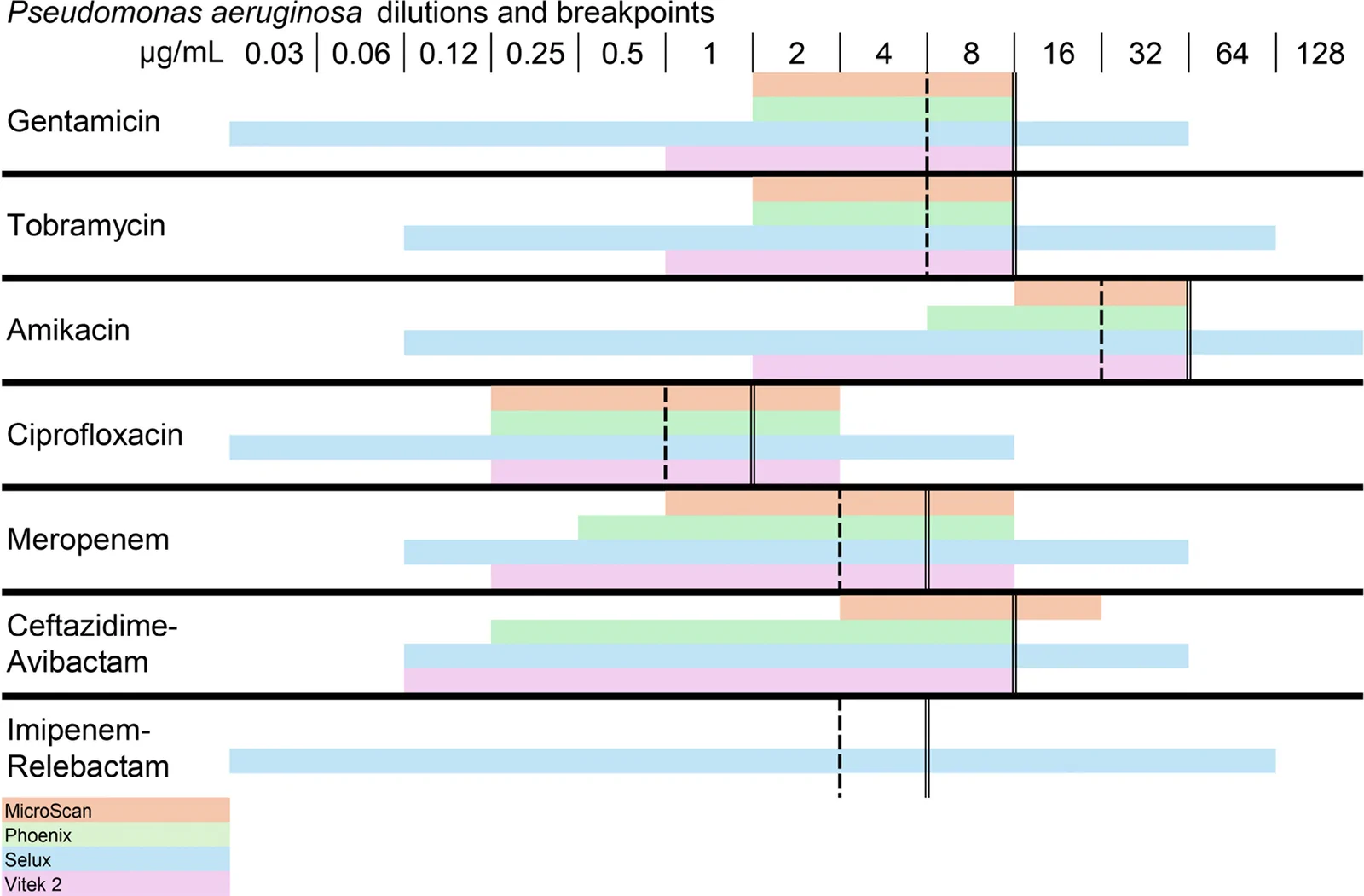
Tested antimicrobial dilutions and breakpoints for *P. aeruginosa.* Dilutions on each panel are depicted on the grid by colored boxes. The 2024 U.S. FDA susceptibility testing interpretative criteria for *P. aeruginosa* are depicted, where the hashed lines demonstrate the breakpoint between susceptible and intermediate categories, and a double solid line demonstrates the breakpoint between resistant and intermediate (or between resistant and susceptible categories).

**TABLE 1:** Gram-negative organisms and sources of isolates evaluated

Organism group	Species/complex	Banked isolates	AR bank isolates^a^	Excluded isolates^b^	Total	Total by organism groups

Enterobacterales	*Citrobacter freundii* complex	15	0	0	15	242
	*Citrobacter koseri*	15	0	0	15	
	*Enterobacter cloacae* complex	20	8	0	28	
	*Escherichia coli*	20	25	0	45	
	*Klebsiella aerogenes*	15	1	1	15	
	*Klebsiella oxytoca*	15	1	0	16	
	*Klebsiella pneumoniae*	20	32	3	49	
	*Morganella morganii*	15	0	0	15	
	*Proteus mirabilis*	15	0	0	15	
	*Proteus vulgaris*	15	0	1	14	
	*Serratia marcescens*	15	0	0	15	
Pseudomonas	*Pseudomonas aeruginosa*	100	33	3	130	130
	Grand total	280	100	8	372	372

a CDC Antimicrobial Resistance Bank.

b Eight isolates were excluded from the data analysis: three clinical isolates and one AR bank isolate that subsequently were determined to have discrepant identities to that which was recorded, one clinical isolate exhibiting multiple morphologies, and three AR bank isolates with BMD results that were discrepant with the CDC-published reference results.

**TABLE 2:** Study sites, systems, and panels for testing

Site	System	Panel(s)

Cleveland Clinic	Vitek 2	GN79 and XN15
Johns Hopkins	Phoenix	NMIC-306 Emerge Panel
Loyola	MicroScan	Neg MIC 56/NM56
Northshore	Disk diffusion	Not applicable
Cleveland Clinic	Selux	Selux GN Panel

**TABLE 3:** Summary of performance of gram-negative bacterial susceptibility testing for multiple commercial systems when applying contemporary interpretive breakpoints^a^

Antimicrobial	Selux NGP		MicroScan		Phoenix		Vitek 2		Disk diffusion
	%EA	%CA	%EA	%CA	%EA	%CA	%EA	%CA	%CA

Amikacin	91.3	94.5	99.2	95.4	96.9	93.8	95.4	96.9	93.8
Amoxicillin-clavulanate	99.2	**86.2**	100	91.1	100	92.7	NA	NA	**73.4**
Ampicillin	98.3	100	100	100	100	98.3	100	100	95.0
Ampicillin-sulbactam	95.0	**79.9**	98.6	**87.0**	98.4	91.2	98.6	**85.6**	**79.9**
Aztreonam	95.6	91.1	**86.4**	**84.1**	97.8	**84.4**	**88.9**	**84.4**	**80.0**
Cefazolin	96.8	96.8	98.9	97.8	100	97.9	NA	NA	93.6
Cefepime	**86.3**	91.7	93.4	90.9	95.0	92.6	**76.8**	**75.9**	**84.3**
Cefoxitin	94.4	**75.2**	98.4	94.4	100	91.2	98.4	**81.6**	**76.0**
Ceftazidime	96.4	93.8	91.2	**89.8**	99.1	96.0	**89.9**	92.1	91.6
Ceftazidime-avibactam	97.3	98.9	100	98.5	98.1	99.2	94.3	98.7	NA
Ceftriaxone	97.6	98.2	97.0	97.6	99.4	99.4	97.1	99.4	97.1
Ciprofloxacin	96.3	95.4	97.8	92.7	93.0	**89.0**	99.4	95.5	95.8
Ertapenem	97.0	98.5	100	98.0	99.5	98.5	98.5	95.5	96.0
Gentamicin	92.8	95.4	96.9	91.9	98.3	94.1	97.8	95.8	95.0
Levofloxacin	93.3	91.6	**83.4**	**89.6**	99.6	93.0	NA	NA	**87.6**
Meropenem	96.6	96.0	98.6	95.5	99.1	96.2	97.8	96.4	95.8
Meropenem-vaborbactam	97.2	98.6	100	98.5	100	98.6	94.4	95.8	96.2
Minocycline	96.6	90.0	94.9	**81.8**	98.0	91.0	95.0	**74.0**	**83**
Piperacillin-tazobactam	91.2	93.2	96.7	94.1	94.1	91.5	97.3	92.7	92.2
Tobramycin	**86.6**	97.6	100	96.9	99.2	98.4	99.2	99.2	98.5
Trimethoprim-sulfamethoxazole	98.1	99.1	100	98.1	99.1	99.1	99.1	99.1	99.1

a Breakpoints recognized in 2024 by the U.S. Food and Drug Administration were applied. Broth microdilution testing in triplicate (median or mode) was used as the comparator reference. As many as 372 isolates of Enterobacterales and *P. aeruginosa* are described in some rows. However, not all antimicrobials are applicable to all isolates or test methods; hence, some rows contain data from fewer than 372 isolates. Bolded text denotes agreement less than 90.0%. NA, not applicable.

**TABLE 4:** Performance of Enterobacterales susceptibility testing of multiple commercial systems when applying contemporary interpretive breakpoints^f^

Antimicrobial^a^	Total isolates^b^				Selux				Micro Scan				Phoenix				Vitek 2				Disk diffusion			
	Total	R	S	I	EA/CA^c^	VMJ^d^	MAJ^d^	MIN^d^	EA/CA	VMJ	MAJ	MIN	EA/CA	VMJ	MAJ	MIN	EA/CA	VMJ	MAJ	MIN	CA	VMJ	MAJ	MIN

AMC	124	46	49	29	99.2/**86.2**	0	0	17 (13.8)	100/91.1	0	0	11 (8.9)	100/ 92.7	0	0	9 (7.3)	NA^e^	NA	NA	NA	**73.4**	0	2 (**4.1**)	30 (24.2)
AMP	60	57	3	0	98.3/100	0	0	0	100/100	0	0	0	100/ 98.3	0	0	1 (1.7)	100/ 100	0	0	0	95.0	1 (1.8)	1 (**33.3**)	1 (1.7)
SAM	139	89	27	23	95.0/**79.9**	0	1 (**3.7**)	27 (19.4)	98.6/**87.0**	0	0	18 (13.0)	98.4/ 91.2	0	0	11 (8.8)	98.6/ **85.6**	0	1 (**3.7**)	19 (**13.7**)	**79.9**	1 (1.1)	1 (**3.7**)	26 (18.7)
ATM	45	31	7	7	95.6/91.1	0	0	4 (8.9)	**86.4/84.1**	0	1 (**14.3**)	6 (13.6)	97.8/ **84.4**	0	0	7 (15.6)	**88.9/ 84.4**	0	0	7 (15.6)	**80.0**	2 (**6.5**)	0	8 (17.8)
CFZ	94	89	2	3	96.8/96.8	0	0	3 (3.2)	98.9/97.8	0	0	2 (2.0)	100/ 97.9	0	0	2 (2.0)	NA	NA	NA	NA	93.6	1 (1.1)	0	4 (4.3)
FEP	242	107	124	11	**86.3**/91.7	0	1 (0.8)	18 (7.5)	93.4/90.9	0	5 (**4.0**)	17 (7.1)	95.0/ 92.6	2 (1.9)	0	16 (6.6)	**76.8/ 75.9**	31 (**29.0**)	1 (0.8)	26 (10.8)	**84.3**	0	0	36 (14.9)
FOX	125	54	70	1	94.4/**75.2**	0	0	11 (8.8)	98.4/94.4	2 (**3.7**)	0	1 (1.0)	100/ 91.2	0	0	11 (8.8)	98.4/ **81.6**	0	0	23 (18.4)	**76.0**	2 (**3.7**)	0	27 (21.6)
CAZ	227	100	120	7	96.4/93.8	1 (1.0)	2 (1.7)	11 (4.9)	91.2/**89.8**	1 (1.0)	4 (**3.3**)	11 (4.9)	99.1/ 96.0	0	0	9 (4.0)	**89.9**/ 92.1	6 (**6.0**)	1 (0.8)	11 (4.8)	91.6	5 (**5.0**)	1 (0.8)	12 (5.3)
CZA	242	22	220	0	96.2/99.6	0	1 (0.5)	0	100/100	0	0	0	97.5/ 99.6	0	0	0	92.9/ 99.6	0	1 (0.5)	0	NA	NA	NA	NA
CRO	170	101	67	2	97.6/98.2	0	0	3 (1.7)	97.0/97.6	0	2 (3.0)	2 (1.2)	99.4/ 99.4	0	0	1 (0.6)	97.1/ 99.4	0	0	1 (0.6)	97.1	1 (1.0)	0	3 (1.8)
CIP	227	123	93	11	96.0/94.2	0	0	13 (5.8)	96.9/92.5	0	4 (**4.3**)	13 (5.8)	95.6/ 92.5	0	2 (2.2)	15 (6.6)	99.1/ 96.0	0	2 (2.2)	7 (3.1)	95.6	2 (1.6)	0	7 (3.1)
ETP	199	44	154	1	97.0/98.5	0	1 (0.7)	2 (1.0)	100/98.0	0	0	4 (2.0)	99.5/ 98.5	0	0	3 (1.5)	98.5/ 95.5	1 (**2.3**)	1 (0.7)	7 (3.5)	96.0	4 (**9.1**)	0	3 (1.5)
GEN	227	78	144	5	96.0/97.3	0	0	6 (2.7)	96.5/94.7	0	5 (**3.5**)	7 (3.1)	98.7/ 96.5	0	1 (0.7)	7 (3.1)	98.2/ 96.5	0	0	8 (3.5)	96.9	1 (1.3)	0	5 (2.2)
LVX	242	117	111	14	93.3/91.6	0	0	20 (8.4)	**83.4/89.6**	1 (0.9)	6 (**5.4**)	18 (7.5)	99.6/ 93.0	0	0	17 (7.0)	NA	NA	NA	NA	**87.6**	6 (**5.1**)	1 (0.9)	22 (9.1)
MEM	226	48	177	1	97.3/97.3	0	1 (0.6)	5 (2.3)	99.6/98.2	0	1 (0.6)	3 (1.3)	99.1/ 98.6	1 (**2.1**)	0	2 (0.9)	98.2/ 98.2	0	2 (1.1)	2 (0.9)	96.5	1 (**2.1**)	0	6 (2.6)
MEV	213	21	190	2	97.2/98.6	0	0	3 (1.4)	100/98.5	0	0	3 (1.5)	100/ 98.6	0	0	3 (1.4)	94.4/ 95.8	0	4 (2.1)	5 (2.3)	96.2	1 (**4.8**)	1 (0.5)	6 (2.8)
MIN	100	14	69	17	96.6/90.0	0	0	9 (10.1)	94.9/**81.8**	1 (**7.1**)	4 (**5.8**)	13 (13.1)	98.0/ 91.0	0	1 (1.4)	8 (8.0)	95.0/ **74.0**	0	0	26 (26.0)	**83.0**	0	1 (14)	16 (16.0)
TZP	153	50	98	5	91.2/93.2	1 (2.0)	3 (**3.2**)	6 (4.1)	96.7/94.1	0	2 (2.1)	7 (4.6)	94.1/ 91.5	0	8 (**8.2**)	5 (3.3)	97.3/ 92.7	0	2 (2.1)	9 (6.0)	92.2	1 (2.0)	4 (**4.1**)	6 (3.9)
SXT	108	67	41	0	98.1/99.1	0	0	0	100/98.1	0	0	0	99.1/ 99.1	1 (1.5)	0	0	99.1/ 99.1	1 (1.5)	0	0	99.1	1 (1.5)	0	0

a Antimicrobial abbreviations: amoxicillin-clavulanate (AMC), ampicillin (AMP), ampicillin-sulbactam (SAM), aztreonam (ATM), cefazolin (CFZ), cefepime (FEP), cefoxitin (FOX), ceftazidime (CAZ), ceftazidime-avibactam (CZA), ceftriaxone (CRO), ciprofloxacin (CIP), ertapenem (ETP), gentamicin (GEN), levofloxacin (LVX), meropenem (MEM), meropenem-vaborbactam (MEV), minocycline (MIN), piperacillin-tazobactam (TZP), and trimethoprim-sulfamethoxazole (SXT).

b Number of isolates reported for total isolates and for resistant (R), susceptible (S), and intermediate (I) as determined by reference testing and FDA breakpoints.

c Essential agreement (EA) and categorical agreement (CA) are reported as a percentage.

d Very major error (VMJ), major error (MJ), and minor error (MIN) are reported as number of isolates (N) and percentage (%) in the format of N (%). Percentages for VMJ were calculated using R as the denominator, and percentages for MAJ were calculated using S as the denominator.

e Not applicable (NA) because not tested.

f Broth microdilution testing in triplicate (median or mode) was used as the comparator reference. Breakpoints recognized in 2024 by the U. S. Food & Drug Administration were applied. Bold text denotes percentages FDA typically considers unacceptable in performance evaluations: EA <90%, CA <90%, VMJ >2%, and MAJ >3%.

**TABLE 5:** Performance of *P. aeruginosa* susceptibility testing of multiple commercial systems when applying contemporary interpretive breakpoints^f^

Antimicrobial^a^	Total isolates^b^				Selux				Micro Scan				Phoenix				Vitek 2				Disk diffusion			
	Total	R	S	I	EA/CA^c^	VMJ^d^	MAJ^d^	MIN^d^	EA/CA	VMJ	MAJ	MIN	EA/CA	VMJ	MAJ	MIN	EA/CA	VMJ	MAJ	MIN	CA	VMJ	MAJ	MIN

AMK	130	12	114	4	91.3/94.5	0	0	7 (5.5)	99.2/95.4	0	0	5 (3.9)	96.9/93.8	2 (**18.2**)	0	6 (4.7)	95.4/96.9	0	0	4 (3.1)	93.8	0	1 (0.9)	7 (5.4)
CZA	130	18	112	0	99.2/97.6	0	0	0	100.0/96.2	0	0	0	99.2/98.4	0	0	0	96.9/96.9	0	1 (0.9)	0	NA^e^	NA	NA	NA
CIP	130	44	82	4	96.9/97.6	0	0	3 (2.4)	99.2/93.1	0	0	9 (7.0)	**88.4/82.9**	2 (**4.7**)	4 (**4.9**)	16 (12.4)	100.0/94.6	0	0	7 (5.4)	96.2	0	0	4 (3.1)
GEN	130	21	99	10	**87.2**/91.2	0	0	11 (8.8)	97.7/**86.9**	0	0	15 (11.5)	97.7/**89.9**	0	0	13 (10.1)	96.9/94.6	0	0	7 (3.8)	91.5	0	2 (2.0)	9 (7.0)
MEM	130	39	85	6	95.3/93.7	0	0	8 (6.3)	96.9/90.8	0	0	12 (9.2)	99.2/92.2	0	0	10 (7.8)	96.9/93.1	0	0	9 (6.9)	94.6	0	0	7 (5.4)
TOB	130	22	106	2	**86.6**/97.6	0	0	3 (2.4)	100.0/96.9	0	0	4 (3.1)	99.2/98.4	0	0	2 (5.4)	99.2/99.2	0	1 (0.9)	0	98.5	0	0	2 (1.5)

a Antimicrobial abbreviations: amikacin (AMK), ceftazidime-Avibactam (CZA), ciprofloxacin (CIP), gentamicin (GEN), meropenem (MEM), tobramycin (TOB).

b Number of isolates reported for total isolates and for resistant (R), susceptible (S), and intermediate (I) as determined by reference testing and FDA breakpoints.

c Essential agreement (EA) and categorical agreement (CA) are reported as a percentage.

d Very major error (VMJ), major error (MJ), and minor error (MIN) are reported as number of isolates (N) and percentage (%) in the format of N (%). Percentages for VMJ were calculated using R as the denominator, and percentages for MAJ were calculated using S as the denominator.

e Not applicable (NA) because not tested.

f Broth microdilution testing in triplicate (median or mode) was used as the comparator reference. Breakpoints recognized in 2024 by the U. S. Food & Drug Administration were applied. Bold text denotes percentages FDA typically considers unacceptable in performance evaluations: EA ≤89.9%, CA ≤89.9%, VMJ >2%, and MAJ >3%.

**TABLE 6:** Percentage bias of the minimum inhibitory concentration determination by multiple commercial test systems when compared to a reference standard^a^

Antimicrobial	Organism group	Selux			MicroScan			Phoenix			VITEK 2		
		*N*	+/−	Amount^b^ (95% CI)	*N*	+/−	Amount (95% CI)	*N*	+/−	Amount (95% CI)	*N*	+/−	Amount (95% CI)

Amikacin	*P. aeruginosa*	127	−	26 (19–35)	8	+	**75 (35–97)**	13	−	69 (39–90)	86	−	49 (38–60)
Amoxicillin-clavulanate	Enterobacterales	123	−	15 (10–23)	33	+	3 (0–16)	60		0	NA		NA
Ampicillin	Enterobacterales	53	−	21 (11–34)	NA		NA	1	−	100 (3–100)	1		0
Ampicillin-sulbactam	Enterobacterales	137	+	13 (5–16)	50	−	38 (25–53)	36	+	31 (16–48)	49		0
Aztreonam	Enterobacterales	45	−	29 (16–44)	14	+	**93 (66–100)**	16	−	38 (15–65)	26	−	54 (33–73)
Cefazolin	Enterobacterales	62	−	16 (8–28)	21	+	**81 (58–95)**	22	+	**68 (45–86)**	22	+	**86 (65–97)**
Cefepime	Enterobacterales	185	+	17 (12–23)	40		0	49	−	27 (15–41)	83	−	83 (73–90)
Cefoxitin	Enterobacterales	120	+	36 (27–45)	13	−	62 (32–86)	40	−	30 (17–47)	70	+	4 (0–12)
Ceftazidime	Enterobacterales	152	+	**52 (44–60)**	55	+	16 (8–29)	40	−	33 (19–49)	64	−	64 (51–76)
Ceftazidime-avibactam	Enterobacterales	225	+	16 (12–22)	4	+	50 (7–93)	88	+	32 (22–43)	118	−	12 (7–19)
	*P. aeruginosa*	123	−	27 (19–36)	20	−	40 (19–64)	114	+	12 (7–20)	114	−	7 (3–13)
Ceftriaxone	Enterobacterales	123	+	2 (0–6)	48	+	**83 (70–93)**	46	+	**87 (74–95)**	51	+	**61 (46–74)**
Ciprofloxacin	Enterobacterales	162	−	9 (5–14)	43	+	**56 (40–71)**	38	+	**92 (79–98)**	38	+	11 (3–25)
	*P. aeruginosa*	122	−	48 (38–57)	27	+	**78 (58–91)**	40	+	**88 (73–96)**	27	+	**63 (42–81)**
Ertapenem	Enterobacterales	163	+	11 (7–17)	4		0	8	+	25 (3–65)	12	+	50 (21–79)
Gentamicin	Enterobacterales	218	−	21 (15–27)	22	+	50 (28–72)	16	+	**88 (62–98)**	21	+	19 (5–42)
	*P. aeruginosa*	123	−	47 (38–56)	63	+	**73 (60–83)**	36	−	53 (35–70)	84	−	43 (32–54)
Levofloxacin	Enterobacterales	233	+	**45 (39–52)**	140	−	72 (64–79)	51	+	28 (16–42)	NA		NA
Meropenem	Enterobacterales	176	+	**86 (80–91)**	13	+	**62 (32–86)**	19	+	16 (3–40)	30	+	57 (37–75)
	*P. aeruginosa*	127	+	**43 (34–52)**	32	+	34 (19–53)	49	+	33 (20–48)	73	+	30 (20–42)
Meropenem-vaborbactam	Enterobacterales	144	+	29 (22–37)	10		0	9	+	22 (3–60)	33	+	**58 (39–75)**
Minocycline	Enterobacterales	71	−	10 (4–19)	26	+	8 (1–25)	78	+	39 (28–50)	88	+	**58 (47–68)**
Piperacillin-tazobactam	Enterobacterales	138	−	2 (0–6)	13	+	15 (2–45)	50	+	**56 (41–70)**	23	+	30 (13–53)
Tobramycin	*P. aeruginosa*	127	−	52 (43–61)	9	+	**100 (66–100)**	5	−	40 (5–85)	25	+	36 (18–57)
Trimethoprim-sulfamethoxazole	Enterobacterales	93	+	4 (1–11)	4	+	50 (7–93)	2	−	50 (1–99)	3	−	33 (0–90)

a Only the minimal inhibitory concentration (MIC) results that are on scale are considered in the U.S. Food and Drug Administration bias calculations, which means “less than or equal to” and “greater than or equal to” MIC results are excluded from bias calculations, and this approach is used in the table.

b Amount of bias is as follows: positive bias (+) indicates the minimum inhibitory concentration (MIC) of the test system trends higher than the reference MIC result and negative bias (−) indicates the MIC of the test system trends lower than the reference MIC result. Text is bold if the lower bound of the 95% confidence interval is ≥30% with positive bias, and text is underlined if the lower bound of the 95% confidence interval is ≥30% with a negative bias. CI, confidence interval; NA, not applicable (because not tested).

**TABLE 7:** Duration of time including isolate setup through results reported by each test system

Test system	*P. aeruginosa*		Enterobacterales	
	Median	95% quantile	Median	95% quantile

Selux	7.25 h	8.54 h	5.78 h	6.55 h
MicroScan	16.20 h	16.38 h	16.28 h	16.55 h
Phoenix	16.28 h	16.85 h	15.18 h	19.22 h
VITEK 2	14.10 h	18.73 h	11.27 h	19.88 h
Disk diffusion	25.08 h	26.55 h	25.67 h	28.25 h

## IMPORTANCE

This study assessed and compared the performance of multiple commercial antimicrobial susceptibility testing (AST) systems and disk diffusion methods. The accuracy of determining minimum inhibitory concentrations or zone diameters of antimicrobials and the associated interpretive categories were studied for 372 isolates of Enterobacterales and *Pseudomonas aeruginosa*. Performance of the evaluated systems revealed that multiple antimicrobial-organism combinations were below the standards typically required by regulatory agencies and clinical laboratories. These deficiencies varied across the commercial platforms and disk diffusion methods, highlighting the importance of benchmarking studies that compare the performance using identical isolates. These identified performance weaknesses may warrant further study and/or the implementation of mitigation strategies in clinical laboratory operations to prevent the reporting of errant results.
