# Supplementary material for: Benchmarking four commercial antimicrobial susceptibility testing systems and disk diffusion to reference broth microdilution in the assessment of gram-negative bacterial isolates
Source: J Clin Microbiol. 2026 Sep 9;64(10):e00502-26. doi: 10.1128/jcm.00502-26 (PMC13650682; doi:10.1128/jcm.00502-26)
Supplement: Supplementary file 1 [file jcm.00502-26-s0001.docx]

**Supplemental Materials**

Supplemental Table 1. Differences between U.S. Food & Drug Administration (FDA) susceptibility test interpretive criteria (STIC) breakpoints used in this study and the CLSI breakpoints at the time the study was performed.

|  |  | **FDA STIC Breakpoints** | | | | **CLSI Break Points**  **(CLSI M100-34th ed)** | | | |
| --- | --- | --- | --- | --- | --- | --- | --- | --- | --- |
| Antimicrobial | Organism Group | **S** | **SDD** | **I** | **R** | **S** | **SDD** | **I** | **R** |
| Cefoxitin | Enterobacterales | ≤4 |  | 8 | ≥16 | ≤8 | - | 16 | ≥32 |
| Gentamicin | Enterobacterales | ≤4 |  | 8 | ≥16 | ≤2 | - | 4 | ≥8 |
| Gentamicin | *Pseudomonas aeruginosa* | ≤4 |  | 8 | ≥16 | NA | NA | NA | NA |
| Piperacillin-Tazobactam | Enterobacterales | ≤8 |  | 16 | ≥32 | ≤8 | 16 |  | ≥32 |
| Tobramycin | *Pseudomonas aeruginosa* | ≤4 |  | 8 | ≥16 | ≤1 | - | 2 | ≥4 |

NA, Not applicable; S, susceptible; SDD, susceptible-dose dependent; I, intermediate; R, resistant

Supplemental Table 2. Antimicrobial-organism combination list for Enterobacterales.*

|  | *C freundii* | *C koseri* | *E cloacae* | *E coli* | *K aerogenes* | *K oxytoca* | *K pneumoniae* | *M morganii* | *P mirabilis* | *P vulgaris* | *S marcescens* |
| --- | --- | --- | --- | --- | --- | --- | --- | --- | --- | --- | --- |
| AMC^a^ |  |  |  |  |  |  |  |  |  |  |  |
| AMP |  |  |  |  |  |  |  |  |  |  |  |
| ATM |  |  |  |  |  |  |  |  |  |  |  |
| CAZ |  |  |  |  |  |  |  |  |  |  |  |
| CFZ |  |  |  |  |  |  |  |  |  |  |  |
| CIP |  |  |  |  |  |  |  |  |  |  |  |
| CRO |  |  |  |  |  |  |  |  |  |  |  |
| CZA^b^ |  |  |  |  |  |  |  |  |  |  |  |
| ETP |  |  |  |  |  |  |  |  |  |  |  |
| FEP |  |  |  |  |  |  |  |  |  |  |  |
| FOX |  |  |  |  |  |  |  |  |  |  |  |
| GEN |  |  |  |  |  |  |  |  |  |  |  |
| LVX |  |  |  |  |  |  |  |  |  |  |  |
| MEM^c^ |  |  |  |  |  |  |  |  |  |  |  |
| MEV^d^ |  |  |  |  |  |  |  |  |  |  |  |
| MIN |  |  |  |  |  |  |  |  |  |  |  |
| SAM^e^ |  |  |  |  |  |  |  |  |  |  |  |
| SXT |  |  |  |  |  |  |  |  |  |  |  |
| TZP |  |  |  |  |  |  |  |  |  |  |  |

*System-specific species exclusions were due to the absence of the antimicrobial from the tested card(s), lack of FDA approval or laboratory-specific limitations that are not system-specific. Shaded boxes were not tested. Non-shaded boxes were tested on all systems except where noted.

^a^AMC. No results AMC from Vitek 2 only. Results available on all other systems.

^b^CZA. No results from MicroScan for *Enterobacter cloacae* and *Proteus vulgaris*. No results from DD.

^c^MEM. No results from Phoenix for *Proteus mirabilis.*

^d^MEV. No results from MicroScan for *Citrobacter freundii* and *Proteus vulgaris*.

^e^SAM. No results from Phoenix for *Proteus vulgaris.*

AMC, amoxicillin-clavulanate; AMP, ampicillin; ATM, aztreonam; CAZ, ceftazidime; CFZ, cefazolin; CIP, ciprofloxacin; CRO, ceftriaxone; CZA, ceftazidime-avibactam; ETP, ertapenem; FEP, cefepime; FOX, cefoxitin; GEN, gentamicin; LVX, levofloxacin; MEM, meropenem; MEV, meropenem-vaborbactam; MIN, minocycline; SAM, ampicillin-sulbactam; SXT, trimethoprim-sulfamethoxazole; TZP, piperacillin-tazobactam

Supplemental Table 3. List of antimicrobials tested on the automated systems, panels used, reported dilutions, and U.S. Food & Drug Administration (FDA) susceptibility test interpretive criteria (STIC) breakpoints.

|  |  | **Selux** | **MicroScan** | **Phoenix** | **Vitek 2** | **FDA STIC Breakpoints**  **(μg/mL)** | | | |
| --- | --- | --- | --- | --- | --- | --- | --- | --- | --- |
| **Antimicrobial** | **Organism Group** | **Reported MIC dilutions (μg/mL)** | | | | **S** | **SDD** | **I** | **R** |
| Amikacin | *Pseudomonas aeruginosa* | ≤0.12 to >128 | ≤16 to >32 | ≤8 to >32 | ≤2 to >32 | ≤16 | - | 32 | ≥64 |
| Amoxicillin-Clavulanate | Enterobacterales | ≤0.5 to >64 | ≤8 to >16 | ≤4 to >16 | *NA* | ≤8 | - | 16 | ≥32 |
| Ampicillin | Enterobacterales | ≤0.25 to >64 | ≤8 to >16 | ≤4 to >16 | ≤2 to >16 | ≤8 | - | 16 | ≥32 |
| Ampicillin-Sulbactam | Enterobacterales | ≤0.5 to >64 | ≤4 to >16 | ≤1 to >16 | ≤2 to >16 | ≤8 | - | 16 | ≥32 |
| Aztreonam | Enterobacterales | ≤0.03 to >64 | ≤4 to >16 | ≤2 to >16 | ≤1 to >32 | ≤4 | - | 8 | ≥16 |
| Cefazolin | Enterobacterales | ≤0.12 to >64 | ≤2 to >16 | ≤1 to>16 | ≤4 to >32 | ≤2 | - | 4 | ≥8 |
| Cefazolin (Urine) | Enterobacterales | ≤0.12 to >64 | ≤2 to >16 | ≤1 to >16 | ≤4 to >32 | ≤16 | - | - | ≥32 |
| Cefepime | Enterobacterales | ≤0.25 to >64 | ≤2 to >16 | ≤1 to >16 | ≤1 to>32 | ≤2 | 4-8 | - | ≥16 |
| Cefoxitin | Enterobacterales | ≤1 to >64 | ≤8 to >16 | ≤4 to>16 | ≤4 to >32 | ≤4 | - | 8 | ≥16 |
| Ceftazidime | Enterobacterales | ≤0.25 to >32 | ≤1 to >16 | ≤2 to >16 | ≤1 to >32 | ≤4 | - | 8 | ≥16 |
| Ceftazidime-Avibactam | Enterobacterales | ≤0.12 to >32 | ≤4 to >16 | ≤0.25 to >8 | ≤0.12 to >8 | ≤8 | - | - | ≥16 |
|  | *P. aeruginosa* |  |  |  |  | ≤8 | - | - | ≥16 |
| Ceftriaxone | Enterobacterales | ≤0.25 to >16 | ≤1 to >32 | ≤1 to >32 | ≤1 to >32 | ≤1 | - | 2 | ≥4 |
| Ciprofloxacin | Enterobacterales | ≤0.03 to >8 | ≤0.25 to >2 | ≤0.25 to >2 | ≤0.25 to >2 | ≤0.25 | - | 0.5 | ≥1 |
|  | *P. aeruginosa* |  |  |  |  | ≤0.5 | - | 1 | ≥2 |
| Ertapenem | Enterobacterales | ≤0.03 to >8 | ≤0.5 to >1 | ≤0.25 to >2 | ≤0.5 to >4 | ≤0.5 | - | 1 | ≥2 |
| Gentamicin | Enterobacterales | ≤0.06 to >32 | ≤2 to >8 | ≤2 to >8 | ≤1 to >8 | ≤4 | - | 8 | ≥16 |
|  | *P. aeruginosa* |  |  |  |  | ≤4 | - | 8 | ≥16 |
| Imipenem-Relebactam | Enterobacterales | ≤0.03 to >64 | *NA* | *NA* | *NA* | ≤1 | - | 2 | ≥4 |
|  | *P. aeruginosa* |  |  |  |  | ≤2 | - | 4 | ≥8 |
| Levofloxacin | Enterobacterales | ≤0.06 to >16 | ≤0.5 to >8 | ≤0.5 to >4 | *NA* | ≤0.5 | - | 1 | ≥2 |
| Meropenem | Enterobacterales | ≤0.12 to >32 | ≤1 to >8 | ≤0.5 to >8 | ≤0.25 to >8 | ≤1 | - | 2 | ≥4 |
|  | *P. aeruginosa* |  |  |  |  | ≤2 | - | 4 | ≥8 |
| Meropenem-Vaborbactam | Enterobacterales | ≤0.06 to >32 | ≤2 to >16 | ≤2 to>16 | ≤0.5 to >32 | ≤4 | - | 8 | ≥16 |
| Minocycline | Enterobacterales | ≤0.25 to >32 | ≤4 to >8 | ≤1 to >8 | ≤0.5 to>16 | ≤4 | - | 8 | ≥16 |
| Piperacillin-Tazobactam | Enterobacterales | ≤0.25 to >256 | ≤8 to >64 | ≤2 to >64 | ≤4 to >64 | ≤8 | - | 16 | ≥32 |
| Tobramycin | *P.aeruginosa* | ≤0.12 to >64 | ≤2 to >8 | ≤2 to >8 | ≤1 to >8 | ≤4 | - | 8 | ≥16 |
| Trimethoprim-sulfamethoxazole | Enterobacterales | ≤0.12 to >16 | ≤0.5 to >2 | ≤0.5 to >2 | ≤1 to >8 | ≤2 | - | - | ≥4 |

MIC, minimum inhibitory concentration; NA, not applicable; Supplemental Table 4. Number of organism-antibiotic tests assessed in the results of this study

Supplemental Table 4. Number of organism-antibiotic tests assessed in the results of this study

| **Antimicrobial** | **Organism group** | **Total isolates** | **Selux** | **MicroScan** | **Phoenix** | **Vitek 2** | **DD** |
| --- | --- | --- | --- | --- | --- | --- | --- |
| Amikacin | *Pseudomonas aeruginosa* | 130 | 127 | 130 | 129 | 130 | 130 |
| Amoxicillin-Clavulanate | Enterobacterales | 124 | 123 | 123 | 124 | NA | 124 |
| Ampicillin | Enterobacterales | 60 | 59 | 59 | 60 | 60 | 60 |
| Ampicillin-Sulbactam | Enterobacterales | 139 | 139 | 138 | 125 | 139 | 139 |
| Aztreonam | Enterobacterales | 45 | 45 | 44 | 45 | 45 | 45 |
| Cefazolin | Enterobacterales | 94 | 94 | 93 | 94 | 38 | 94 |
| Cefepime | Enterobacterales | 242 | 238 | 241 | 242 | 241 | 242 |
| Cefoxitin | Enterobacterales | 125 | 125 | 124 | 125 | 125 | 125 |
| Ceftazidime | Enterobacterales | 227 | 225 | 226 | 227 | 227 | 227 |
| Ceftazidime-Avibactam | Enterobacterales | 242 | 239 | 199 | 242 | 240 | NA |
|  | *P. aeruginosa* | 130 | 125 | 130 | 129 | 130 | NA |
| Ceftriaxone | Enterobacterales | 170 | 169 | 169 | 170 | 170 | 170 |
| Ciprofloxacin | Enterobacterales | 227 | 224 | 226 | 227 | 226 | 227 |
|  | *P. aeruginosa* | 130 | 127 | 130 | 129 | 130 | 130 |
| Ertapenem | Enterobacterales | 199 | 197 | 198 | 199 | 198 | 199 |
| Gentamicin | Enterobacterales | 227 | 223 | 226 | 227 | 227 | 227 |
|  | *P. aeruginosa* | 130 | 125 | 130 | 129 | 130 | 130 |
| Levofloxacin | Enterobacterales | 242 | 239 | 241 | 242 | NA | 242 |
| Meropenem | Enterobacterales | 226 | 221 | 226 | 212 | 226 | 226 |
|  | *P. aeruginosa* | 130 | 127 | 130 | 129 | 130 | 130 |
| Meropenem-Vaborbactam | Enterobacterales | 213 | 211 | 197 | 213 | 213 | 213 |
| Minocycline | Enterobacterales | 100 | 89 | 99 | 100 | 100 | 100 |
| Piperacillin-Tazobactam | Enterobacterales | 153 | 148 | 152 | 153 | 150 | 153 |
| Tobramycin | *P. aeruginosa* | 130 | 127 | 130 | 129 | 130 | 130 |
| Trimethoprim-sulfamethoxazole | Enterobacterales | 108 | 106 | 108 | 108 | 108 | 108 |
| **Total Tested** | **All** | **3943** | **3872** | **3869** | **3909** | **3513** | **3571** |
